# Urinary incontinence and risk of functional decline in older women: data from the Norwegian HUNT-study

**DOI:** 10.1186/1471-2318-13-47

**Published:** 2013-05-16

**Authors:** Ragnhild Omli, Steinar Hunskaar, Arnstein Mykletun, Ulla Romild, Esther Kuhry

**Affiliations:** 1 Department of Medicine, Division of Geriatrics, Nord-Trøndelag Health Trust, Namsos Hospital, Namsos, Norway; 2 Research Group for General Practice, Department of Public Health and Primary Health Care, University of Bergen, Bergen, Norway; 3 Department of Public Mental Health, Norwegian Institute of Public Health, Division of Mental Health, Oslo, Norway; 4 Faculty of Psychology, University of Bergen, Bergen, Norway; 5 Department of Health Sciences, Nord-Trøndelag Health Trust, Namsos Hospital, Namsos, Norway; 6 Swedish National Institute of Public Health, Østersund, Sweden; 7 NSALK, Department of Surgery, St. Olavs Hospital, Trondheim University Hospital, Trondheim, Norway

## Abstract

**Background:**

The main objective of the present study was to determine whether UI is an independent predictor of ADL decline and IADL decline in elderly women. We also aimed to find out whether incontinent subjects were at higher risk of needing help from formal home care or home nursing care during 11 year follow-up.

**Methods:**

A prospective cohort study conducted as part of the North-Trøndelag Health Study 2 and 3. Women aged 70–80 years when participating in the HUNT 2 study, who also participated in the HUNT 3 study, were included in this study. Analyses on self-reported urinary incontinence at baseline and functional decline during a11-year period were performed for incontinent and continent subjects.

**Results:**

Baseline prevalence of urinary incontinence was 24%. At on average eleven year follow up, logistic regression analysis showed a significant association between incontinence and decline in activities of daily living (ADL) (OR =2.37, 95% CI =1.01-5.58) (*P*=0.04). No association between urinary incontinence and instrumental activities of daily living (IADL) in incontinent women compared with continent women was found (OR=1.18, CI=.75-1.86) (P=.46). Data were adjusted for ADL, IADL and co morbid conditions at baseline. No significant differences in need of more help from formal home care and home nursing care between continent and incontinent women were found after 11 years of follow-up.

**Conclusions:**

Urinary incontinence is an important factor associated with functional decline in women aged 70–80 years living in their own homes. At eleven years of follow up, no significant differences in need of more help from formal home care and home nursing care between continent and incontinent women were found.

## Background

Urinary incontinence (UI) affects 15 to 50% of community-dwelling older women [1-3]. It is defined by the International continence society as “the complaint of any involuntary loss of urine” [4]. In the elderly population, UI is common and often part of a complex condition that occurs due to age-related changes in the lower urinary tract system and medical conditions. Due to the higher occurrence of diseases, such as for example diabetes and cerebral vascular diseases, elderly people often use several types of medication that can disturb the function of the urinary bladder and the continence mechanisms [5].

In the literature, UI is mentioned as one of the geriatric syndromes, together with pressure ulcers, functional decline, falls, and delirium [6]. Previous studies in community-dwelling older people have suggested that the disability to perform Activities of Daily Living (ADL) and Instrumental Activities of Daily Living (IADL) may be associated with new onset urinary incontinence in the older population [7-11]. In a 1- day survey among incontinent elderly receiving home care services, most of the participants had one or more functional disabilities [8]. Early detection of people who are at risk for losing their autonomy is important, so programs can be started to try to prevent or delay functional decline. Hébert suggest that UI should be evaluated as a risk factor for loss of functional independency [12]. Both the feeling of losing control over ones bladder function and independency in ADL and IADL might cause reduced quality of life, depression and social isolation [13,14].

Most studies involve older people that live in nursing homes, residential care homes and hospitals. Few studies have examined urinary incontinence in community-dwelling older people living in their own homes [7,8].

The main objective of the present study was to determine whether UI was an independent predictor in ADL decline and IADL decline during an eleven year follow up period in women aged 70–80 years at baseline, living in their own homes. We also aimed to find out whether incontinent subjects were at risk of needing home care or home nursing care.

## Methods

The Nord-Trøndelag Health study (The HUNT Study) is a cross-sectional study performed in the Nord- Trøndelag County in Norway. This county is fairly representative of the whole nation with respect to geographics, occupation of inhabitants and demographics. The county consists of 24 municipalities with populations ranging from less than 1000 to about 21000 inhabitants [15]. The HUNT study has been performed in three periods. HUNT 1 was conducted during 1984–86, HUNT 2 during 1995–97 and HUNT 3 during 2006–08. All inhabitants aged 20 years or older residing in Nord-Trøndelag during each of these periods were invited to participate.

All participants received an invitation letter, an information folder and a comprehensive questionnaire, which they filled out at home and returned by mail. They were also invited to a screening station where a health team performed physical tests, like height and weight, blood pressure, heart rate and blood sampling [15]. The survey covered many topics including health, personal environment, personal habits and medical history. Regarding medical diagnoses, the participants answered the following question: “Has a doctor ever said that you have/have had any of these diseases:” Myocardial infarction, stroke, diabetes, rheumatoid arthritis, arthritis, hip fracture, depression, hysterectomy? At the screening station, the respondents filled in another questionnaire that included the sub study EPINCONT (EPidemiology of INCOntinence in the county of Nord-Trøndelag), which is a cohort study that focuses on urinary incontinence. The response rate on these questions in women aged from 60–80 years was 69% [3].

The present study is part of the Norwegian North-Trøndelag Health Survey part 2 and 3 (HUNT 2 and HUNT 3). Women aged 70–80 years who participated in both HUNT 2 and HUNT 3 and who answered the EPINCONT questionnaire were included in the analyses. The mean period of follow-up for the participants was 11 (± 2) years.

### Urinary incontinence

Urinary incontinence was defined as any leakage. The entry question: “Do you experience involuntary loss of urine?” was followed by a question about frequency of incontinence (four levels) and amount of urinary loss (three levels). Participants answering “yes” on the entry question and participants answering “yes” on frequency, volume and type of incontinence despite failing to confirm the entry question, was included in the incontinence group [3].

### Functional decline

Both at the HUNT 2 (baseline) and at the HUNT 3 (follow-up) the participants were asked whether they were dependent on help to perform activities of daily living (ADLs) and instrumental activities of daily living (IADLs). In the present study, the following questions regarding ADL-functions was asked: “Do you, without daily help, manage to: eat on your own, go to bed/stand up, dress yourself, go to the lavatory on your own and take a bath or a shower on your own?” The questions regarding IADL were: “Do you manage the following activities without help: shopping/errands, preparing hot meals, taking medications, light housework, heavy housework?” Alternatives for answers were “yes”, “with some help”, or “no.” These were coded as “yes” = manage, “with some help/no” = need help. ADL and IADL were retrieved from HUNT 2 and HUNT 3.

Functional decline was defined as a decline in the ADL or IADL score in the period between HUNT 2 and HUNT 3. The ability to perform ADL and IADL when participating in HUNT 2 was measured by the sum of the ADL-items and IADL-items the participants did not need help to perform. Analyses on dependency in ADL and IADL functions during an 11-year period were performed for incontinent and continent subjects.

The participants were also asked whether they needed help from formal home care help and /or home nursing care to perform ADL and IADL in both HUNT 2 and HUNT 3. In order to receive formal home help services in Norway, the need of help has to be validated by nurses from the public home services.

### Ethics

The HUNT study was approved by the Regional Committee for Medical Research Ethics and the Data Inspectorate of Norway. All data were treated according to the guidelines of the Norwegian Data Inspectorate. Participation in the study was voluntary, and all participants signed a written consent prior to participation both in HUNT 2 study and in the HUNT 3 study. They were also informed about the right to withdraw from the study at any time. Approval from the Data Inspectorate was obtained before data from the HUNT database were retrieved. The study complies with ethical rules for human experimentation as stated in the Declaration of Helsinki [16].

### Statistical analysis

All statistical analyses were performed using PASW Statistics 18 (SPSS18) software (IBM Corp). The main objective of the study was to determine whether UI was an independent predictor of ADL decline and IADL decline. Pearson’s Chi-Square Tests were used to compare baseline differences in morbidity and disease in incontinent and continent women.

Bivariate logistic regression analyses were conducted to assess the unadjusted associations between UI and the decline of ADL and IADL respectively. The models were then adjusted for ADL or IADL at baseline and furthermore adjusted for age, physical impairment and diseases at baseline (diabetes, myocardial infarction, stroke, arthritis and depression), with one model per confounder and outcome, and a final model with all confounders included for the decline of ADL and IADL respectively. The decline in ADL/IADL functions was used as dependent variables in the regression models. The status of ADL was computed as the number of ADL items not functioning at HUNT 2 and at HUNT 3. Decline was defined as an increase in the number of not functioning items from HUNT 2 to HUNT 3. The decline in IADL was computed accordingly. The decline variable is thus dichotomous. The nature of the different items varies, and the number of increased items is not a continuous variable and could not be used as such. Declined ADL was defined as at least one not functioning item and need for help. The decline during follow-up was defined as the difference between HUNT 2 and HUNT 3. Since both the status at HUNT 2 and the decline are dichotomous there should not be any multicollinearity problem. The status at HUNT 2 was used as an explanatory factor and the decline was the dependent variable.

## Results

Of the 6152 women aged 70–80 years who were invited to participate in HUNT 2, 4660 (75%) answered the registration forms and met at the screening station. A total of 3355 out of 4660 (72%) answered the EPINCONT questionnaire. Of those, 770 participated in both HUNT 2 and HUNT 3. These participants were included in the current study. The mean age of the included participants in HUNT 2 and HUNT 3 was 73 years (range 70–80) and 84 years (range 83–92), respectively. Characteristics and co morbidity of the participants in HUNT 2 and HUNT 3 are shown in Table 1. Urinary incontinence was reported by 183 (24%) of the women in HUNT 2. When participating in HUNT 3, 353 women (46%) confirmed that they had UI (P<0.001). No significant differences in ADL or IADL at baseline between continent and incontinent women were found.

**Table 1:** Patient characteristics when participating in HUNT 2 and HUNT 3

**Characteristic**	**Urinary incontinence**			**Urinary incontinence**		
	**HUNT 2**			**HUNT 3**		
	**Yes 183 (24)**	**No 587 (76)**	**P-value**	**Yes 353 (46)**	**No 417 (54)**	**P-value**
Age, mean ± SD	73 ± 2.8	73 ± 2.6		84±2.3	84 ± 2.6	
Physical impairment	26 (14)	63 (10)	.199	69 (38)	203 (35)	.440
Myocardial infarction	8 (4)	19 (3)	.466	17 (9)	47 (8)	.587
Stroke	7 (4)	12 (2)	.175	18 (10)	41 (7)	.208
Diabetes	8 (4)	20 (3)	.543	19 (10)	52 (9)	.538
Rheumatoid arthritis	6 (3)	18 (3)	.885			
Arthritis	68 (37)	159 (27)	.009			
Hip fracture	7 (4)	22 (4)	.962	15 (8)	40 (7)	.526
Depression	8 (6)	21 (5)	.599	8 (6)	15 (3)	.227
Hysterectomi	22 (12)	70 (12)	.972	27 (15)	83 (14)	.836
Consulted physician	153 (88)	430 (78)	.005	83 (51)	60 (14)	<001
Treatment UI				60 (32)	43 (7)	<001

Numbers are given as n(%). Participants often had more than one disease.

In the regression analysis, urinary incontinence was significantly associated with ADL-decline in older women during an eleven-year period. Women who were incontinent were more than twice as likely to experience decline in ADL during the follow-up (OR =2.37, 95% CI =1.01-5.58, *P*=.04) compared to continent women. No significant association between urinary incontinence and IADL-decline was found (OR=1.18, CI=.75-1.86, *P*=.46). Also, ADL-decline at baseline and certain types of diseases were associated with decline in ADL and IADL (see Table 2). At baseline, more incontinent women needed help from formal home care help services and/or home nursing care to perform activities of daily living and instrumental activities of daily living, respectively 19.5% in incontinent women and 11.2% in continent women (*P*<.01). At follow-up however, there were no significant differences between the two groups.

**Table 2:** Urinary incontinence and decline in ADL and IADL activities during 10 year follow-up

**Variable**	**ADL**	**IADL**
	**Odds ratio (95% confidence interval)**	
Incontinence, crude	1.93 (.98-3.82)	1.28 (.88-1.85)
Incontinence, adjusted	2.37 (1.01-5.58)	1.18 (0.75-1.86)
Age at participating	1.17 (1.05-1.31)	1.21 (1.06-1.19)
ADL/IADL	5.65 (1.47-21.77)	0.26 (0.11-.62)
Physical impairment	1.17 (0.44-3.11)	1.86 (0.79-2.10)
Diabetes	0 .64 (0.08-4.96)	1.32 (0.58-2.98)
Myocardial infarction	1.33 (0.29-6.01)	1.04 (0.42-2.56)
Stroke	2.50 (.52-11.91)	1.40 (0.51-3.82)
Arthritis	0.73 (.06-5.63)	1.40 (0.58-3.37)
Depression	0.88 (.11-6.96)	1.23 (0.50-3.04)

Adjusted for potential confounders.

## Discussion

The presence of UI might be of prognostic importance for loss of autonomy and for functional decline. The main finding in this longitudinal study confirms UI to be a significant risk factor for ADL-decline and that UI is not associated with decline in IADL-functions in homebound older women over a period of eleven years, after adjusting for baseline ADL and IADL decline, age, physical impairment and co morbidity. The results of the study confirm that UI is a risk factor for ADL-decline. However, no effect on the risk of needing home care was verified. This might be explained by the possibility that the declined women received informal help from spouse or other relatives. Unfortunately, we did not have data to verify this. In a recent Belgian study, results showed that there might be more use of informal caregivers in municipalities with few inhabitants. In North Trøndelag, most of the municipalities have less than 5000 inhabitants. In a study among frail elderly with UI in Japan, the need of professional care and informal care was studied. Incontinent subjects were grouped into three levels of severity. Those who were in the moderate incontinence group received most help from their informal caregiver [17].

Huang et al. studied decline in physical function and urinary incontinence in community-dwelling women aged 65 and older. They measured the time used for walking 6 meters and for completing five chair stands to define decline in physical function, and found that decline in physics explained an estimate of 9% of clinically frequent incontinence [18]. Results from a Canadian cross sectional study showed that need of help to perform ADL and IADL can mainly be explained by the presence of chronic conditions. Their results also showed a relation between urinary incontinence and ADL-dependency (OR 1.65) and IADL-dependency (OR 1.70) [19].

After the eleven years of follow up there were no differences in the need of help from formal home care and home nursing care. An overall rise in the prevalence of UI and co-morbidity might explain this. Characteristics of the participants showed no statistical differences between the presence of diseases in incontinent and continent participant, either when participating in HUNT 2 or in HUNT 3.

UI was defined as presently having “any involuntary loss of urine”. Twenty-four percent of the participating women answered “yes” on the entry question and/or on questions regarding severity and type of incontinence. This prevalence is in agreement with findings of other studies [9,20-22]. After eleven years of follow up however, 353 (46%) of the women reported that they had UI. The large increase in UI might be explained both by an increase in physical impairment and in the increased prevalence of diseases like diabetes and stroke, which are known to be risk factors for UI in the elderly.

Many of the participants in the present study had one or more diseases associated with functional decline. The relation between UI, stroke and functional decline seems to be quite complex. Some studies suggest that UI is a marker for both stroke severity and disability [23-25].

An increased risk for developing UI at least one year after diagnosis of diabetes 2 was found in a prospective, observational study among elderly women [26]. Ebbesen et al. found severe UI to be associated with diabetes drug treatment [27]. A longitudinal study among women aged 65 years and older showed that diabetic women had a lower IADL score and had more problems with walking [28]. Arthritis which can affect mobility and ability to perform ADL and IADL, were significantly more common in incontinent women at baseline in the HUNT study. Unfortunately, we did not have data on arthritis at follow-up. Turner-Stokes found that rheumatism or arthritis was present in 47.3% of older people ≥ 65 living in their own homes and that these conditions were significantly related to dependency in ADL and IADL [29]. In some cases, physical impairment can cause UI [2,19]. This is often referred to as functional incontinence.

However, the results emphasizing the complexity related to UI in elderly women with both co-morbidity and dependency in performing ADLs and IADLs. Lately there has been more focus on UI as part of a Geriatric syndrome and that there is need of a more comprehensive and broader focus when investigating and treating UI in the elderly [6].

### Strengths and limitations

The longitudinal design with an eleven year follow up adds knowledge to understand the importance of UI as a risk factor for decline in ADL-functions in the population of elderly women. The results also highlight the complexity and the multi factorial etiology of UI in this population that must be taken to account when validating and treating urinary incontinence in older women. The study is based on self-reported data from the participants, which might be a limitation. However, other authors have found that community dwelling older people`s validation of their own health to predict functional decline is valuable [30].

We did not perform an analysis on concomitant diseases and we did not have any data on dementia and Parkinson`s disease, which could represent a limitation. There is a lack of a standardized measurement of functional decline, which might cause difficulties when comparing different studies.

## Conclusions

Urinary incontinence is an important factor associated with functional decline in women aged 70–80 years living in their own homes during eleven years of follow up. Many of the participants also had one or more diseases that are known to be risk factors both to UI and functional decline, which makes UI a highly prevalent part of a multi factorial condition.

After eleven years of follow up, no significant differences in the need of help from formal home care and home nursing care between continent and incontinent women were found.

## Competing interests

None of the authors disclosed any conflicts of interest.

## Authors’ contributions

RO designed and wrote the protocol of the study, contributed to the analyses and interpretation of the data and authored the manuscript. SH designed the EPINCONT study as a part of The HUNT 2 study, and contributed with acquisition of the data and conception of design of the article. AM supervised design, analysis and data interpretation. UR contributed with the statistical analysis and the interpretation of the data. EK revised the article critically for intellectual content and contributed to interpretation of the analysis. All authors made a final approval of the version to be published.

## Pre-publication history

The pre-publication history for this paper can be accessed here:

http://www.biomedcentral.com/1471-2318/13/47/prepub
